# Tai Chi for Improvement of Motor Function, Balance and Gait in Parkinson's Disease: A Systematic Review and Meta-Analysis

**DOI:** 10.1371/journal.pone.0102942

**Published:** 2014-07-21

**Authors:** Yan Yang, Xiang-Yuan Li, Li Gong, Yun-Liang Zhu, Yan-Lei Hao

**Affiliations:** Department of Neurology, Affiliated Hospital of Jining Medical University, Jining, Shandong, China; Cardiff University, United Kingdom

## Abstract

**Background:**

Recently, several studies assessed the effectiveness of Tai Chi for Parkinson's disease (PD), but the role of Tai Chi in the management of PD remained controversial. Therefore, the purpose of this systematic review is to evaluate the evidence on the efficacy of Tai Chi for PD.

**Methods:**

Six English and Chinese electronic databases, up to April 2014, were searched to identify relevant studies. The risk of bias in eligible studies was assessed by Cochrane Collaboration's tools. The primary outcomes were motor function, balance and gait in individuals with PD. Standardized mean difference (SMD) and 95% confidence intervals (CI) of random-effect model were calculated. And heterogeneity was assessed based on the *I^2^*statistic.

**Results:**

7 randomized controlled trials and 1 non-randomized controlled trial were eligible. The aggregated results suggested that Tai Chi showed beneficial effects in improving motor function (SMD, −0.57; 95% CI −1.11 to −0.04; p = 0.03), balance (SMD, 1.22; 95% CI 0.80 to 1.65; p<0.00001) and functional mobility (SMD, 1.06; 95% CI 0.68 to 1.44; p<0.00001) in patients with PD, but not in improving gait velocity (SMD, −0.02; 95% CI −0.58 to 0.54; p = 0.94), step length (SMD, −0.00; 95% CI −0.57 to 0.56; p = 0.99), or gait endurance (SMD, 0.53; 95% CI −0.07 to 1.12; p = 0.08). Comparing with other active therapies, however, Tai Chi only showed better effects in improving balance (SMD, 0.74; 95% CI 0.38 to 1.10; p<0.0001).

**Conclusion:**

Tai Chi should be a valid complementary and alternative therapy for PD, especially in improving motor function and balance. However, more studies with long follow-up are warrant to confirm the current finding of Tai Chi for PD.

## Introduction

Parkinson's disease (PD), a common neurodegenerative disorder, has long been recognized by its cardinal clinical symptoms such as bradykinesia, rigidity, rest tremor, balance disruption and gait impairment [1]. These are important determinants of motor function disability and lower quality of life. It is estimated globally that prevalence of PD is 0.3% in the general population and 1%–2% in persons 60–65 years and older [2], [3]. In China, PD prevalence is estimated as 1.70% in people aged more than 65 years old [4]. In order to seek for alleviation, patients with PD often turn to complementary and alternative medicine [5].

Tai Chi, as a Chinese traditional mind-body exercise, becomes a popular form of complementary and alternative medicine with similarities to aerobic exercise worldwide. When practicing Tai Chi, body movements are integrated with deep diaphragmatic breathing, which can balance body and mind and facilitate the flow of internal energy (Qi of traditional Chinese medicine) [6]. Recent studies reported that Tai Chi might be safe and effective for patients with PD [7]. A previous research also has substantiated that Tai Chi can improve balance, kinesthetic sense and strength, hence it may be prescribed as a sensorimotor agility program for patients with PD [8]. However, the findings related to the motor function and task performances were inconsistent with these studies. Li et al. reported that Tai chi training appeared to reduce balance impairment in patients with mild-to-moderate PD, with additional benefits of improved functional capacity and reduced falls. [9]. Compared with this study, Amano et al. reported that Tai Chi was ineffective in improving either gait dysfunction or reducing parkinsonian disability [10].

Therefore, the purpose of this study was to summarize and evaluate the evidence on the efficacy of Tai Chi for PD. We performed the meta-analyses of Tai Chi for PD, especially on motor function, balance and gait in individuals with PD.

## Methods

### Search Strategy

The following electronic databases were searched from their inceptions up to April 2014: PubMed, EMBASE, Cochrane Library and three Chinese databases (China Knowledge Resource Integrated Database, Weipu Database for Chinese Technical Periodicals and Wan Fang Data). The following search terms were used in combinations: Parkinson's disease, Parkinson, Tai Chi, taiji and shadowboxing. In order to include unpublished studies, dissertations (ProQuest Dissertations and Chinese Dissertation Full-text Database) and trial registrations (WHO International Clinical Trials Registry Platform) were also searched. Further, all reference lists of located articles were searched manually for additional eligible studies. The literature search was carried out independently by two authors (X Li and Y Zhu), and any disagreement was settled by discussion.

### Study Selection

The eligible studies must meet the following criteria: (1) study design: randomized controlled trials (RCTs) and non-randomized controlled trials (non-RCTs); (2) types of participants: participants were diagnosed with PD; (3) types of interventions: Tai Chi compared with placebo, no intervention and any other therapies; Tai Chi combined with conventional drugs compared with conventional drugs or other therapies combined with conventional drugs (e.g. madopar) (4) types of outcomes: motor function, balance and gait; (5) the paper was available in either English or Chinese. The studies were excluded if: (1) the studies were reported without detailed information of primary outcomes; (2) the outcomes of the first phase could not be extracted in the cross-over studies.

Two authors (X Li and Y Zhu) independently screened the titles and abstracts of identified articles. And the full text classified as included and unclear studies was assessed by inclusion and exclusion criteria. The authors resolved all disagreements by discussion.

### Data Extraction

Two authors (Y Yang and L Gong) independently extracted data from included studies based on a pre-designed data extraction form. The following information was extracted: general information (first author, year of publication and country performed studies); study characteristics (study design, patients number, mean age, and Hoehn and Yahr stage), details of the interventions (style and form of Tai Chi, Tai Chi dosage, treatment duration and comparison details) and main outcome measures assessing motor function, balance and gait. The authors of eligible studies were contacted for incompletely reported data. And the disagreements between the reviewers were resolved by discussion.

### Risk of Bias

The risk of bias was assessed independently by two authors (Y Yang and X Li) using Cochrane Collaboration's tools with the following domains: random sequence generation, allocation concealment, blinding of outcome assessment, incomplete outcome data, selective reporting and other bias. In assessing blinding methods, the reviewers paid close attention to blinding of outcome assessment because it was difficult to blind patients and therapists in the non-pharmacological clinical trials. And blinding assessors is crucial to fix these failings. Every domain could be classified as high, low and unclear risk of bias according to the criteria defined in the Cochrane Handbook for Systematic Reviews of Interventions [11]. There was no disagreement between the authors regarding risk of bias in eligible studies.

### Statistical Analysis

The data of the outcome in eligible studies was combined in the meta-analysis using Cochrane Collaboration software (Rev Man 5.1). Standardized mean difference (SMD) and 95% confidence intervals (CI) were calculated in the meta-analyses. The pooled estimate of efficacy was calculated using more conservative random-effect model because various styles of Tai Chi were used in included studies. The *I^2^*statistic was employed in assessing heterogeneity. Heterogeneity was regarded high when the *I^2^* was >75%. Detailed subgroup analyses were conducted based on different types of comparisons and outcome measures.

## Results

### Literature Search

**Figure 1:**
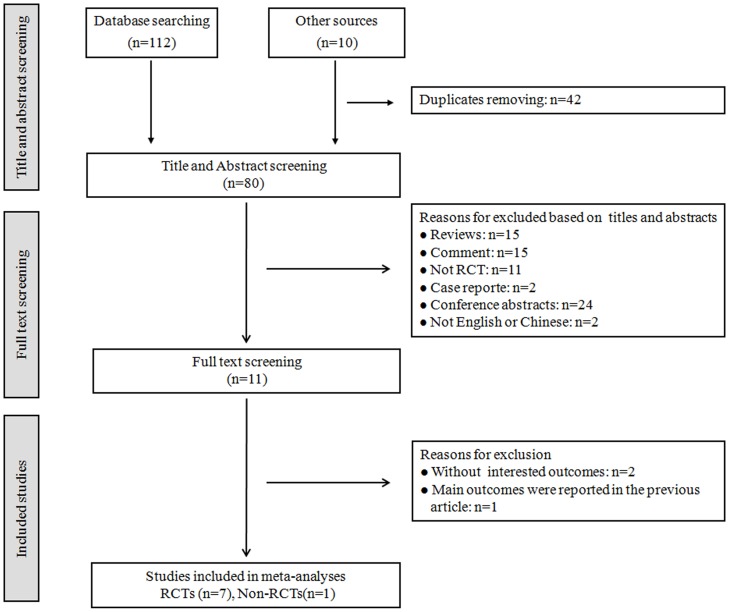
Study selection process. RCTs: randomized controlled trials, Non-RCTs: non-randomized controlled trials.

The initial search retrieved 122 relevant records. After eliminating duplicates, the number of relevant records was reduced to 80, among which 69 records were excluded from the review based on various reasons. And the full texts of 11 studies were screened. 7 RCTs [9], [10], [12]–[16] and 1 non-RCT [17] were included in our review. 6 studies were published in English [9], [10], [12], [15]–[17] and 2 in Chinese [13], [14]. The detailed process of selecting studies was summarized in Figure 1.

### Study Characteristics

The characteristics of 8 included studies were summarized in Table 1. The studies were published in China, Korea and US between 2008 and 2014. The sample size of included studies ranged from 20 to 195 (470 in total), and mean age ranged from 63 to 69. Most studies included patients with mild-to-moderate PD including 6 with Hoehn and Yahr stage I to III [10], [12]–[15], [17] and 2 with Hoehn and Yahr stage I to IV[9], [16].

**Table 1:** Characteristics of the studies included in the meta-analyses.

Study ID	Study design	Patients No.; Mean age	Hoehn and Yahr stage	Study group (n)	TC form or style	Protocol	Main outcomes
Hackney, 2008, US [12]	RCT	26;64	1.5–3	TC (13); No intervention (13)	Yang-style TC	20 times/10–13 wk; 60 min/per time	UPDRS III, BBS, Gait, TUG,6-MWD,TST,OLST
Li, 2011, China [13]	RCT	47;68	2.5–3	TC (24); Walking (23)	24-short form TC	8 wk×10 times/wk; 40 min/per time	UPDRSIII, BBS
Zhu, 2011, China [14]	RCT	38;64	1–2	TC (19); Walking (19)	24-short form TC	4 wk×10 times/wk; 40 min/per time	UPDRSIII, BBS
Li, 2012, US [9]	RCT	195;69	1–4	TC (65); Stretching (65); Resistance training (65)	6 TC movements	24 wk×2 times/wk; 60 min/per time	UPDRS III, Gait, TUG, FRT
Amano, 2013, US [10]	RCT	45;66	2–3	TC (12/15); Qigong (9); No intervention (9)	Yang-style TC	32–48 times/20 wk; 60 min/per time	UPDRS III, Gait
Choi, 2013, US [15]	RCT	20;63	1–2	TC (11); No intervention (9)	NR	12 wk×3 times/wk; 60 min/per time	UPDRS, TUG, Gait, 6-MWD
Gao, 2014, China [16]	RCT	76;69	1–4	TC (37); No intervention (39)	Yang-style TC	12 wk×3 times/wk; 60 min/per time	UPDRSIII, BBS, TUG
Cheon, 2013, Korea [17]	Non-RCT	23;64	2–3	TC (9); Exercise (7); No intervention (7)	Sun-style TC	8 wk×3 times/wk; 50–65 min/per time	UPDRS

No. =  number; TC =  Tai Chi; RCT =  randomized controlled trial; wk =  week; UPDRS =  unified Parkinson's disease rating scale; BBS =  berg balance scale; TUG =  timed up and go; 6-MWD =  6-minute walking distance; TST =  tandem stance test; OLST =  one leg stance test; FRT =  functional-reach test; NR =  no reported; Non-RCT =  non-randomized controlled trial.

The style of Tai Chi included Yang-style Tai Chi, Sun-style Tai Chi, 24-short form Tai Chi, etc. The comparisons included no intervention, walking, stretching/resistance training, Qigong and other exercises. The treatment duration ranged from 4 weeks to 24 weeks. Motor function in patients with PD was assessed by Unified Parkinson's Disease Rating Scale III (UPDRS III), balance by the Berg Balance Scale (BBS), tandem stance test (TST), one leg stance test (OLST) and functional-reach test (FRT), functional mobility by Timed Up and Go test (TUG), gait endurance by 6-minute walk distance (6-MWD).

### Risk of Bias

**Figure 2:**
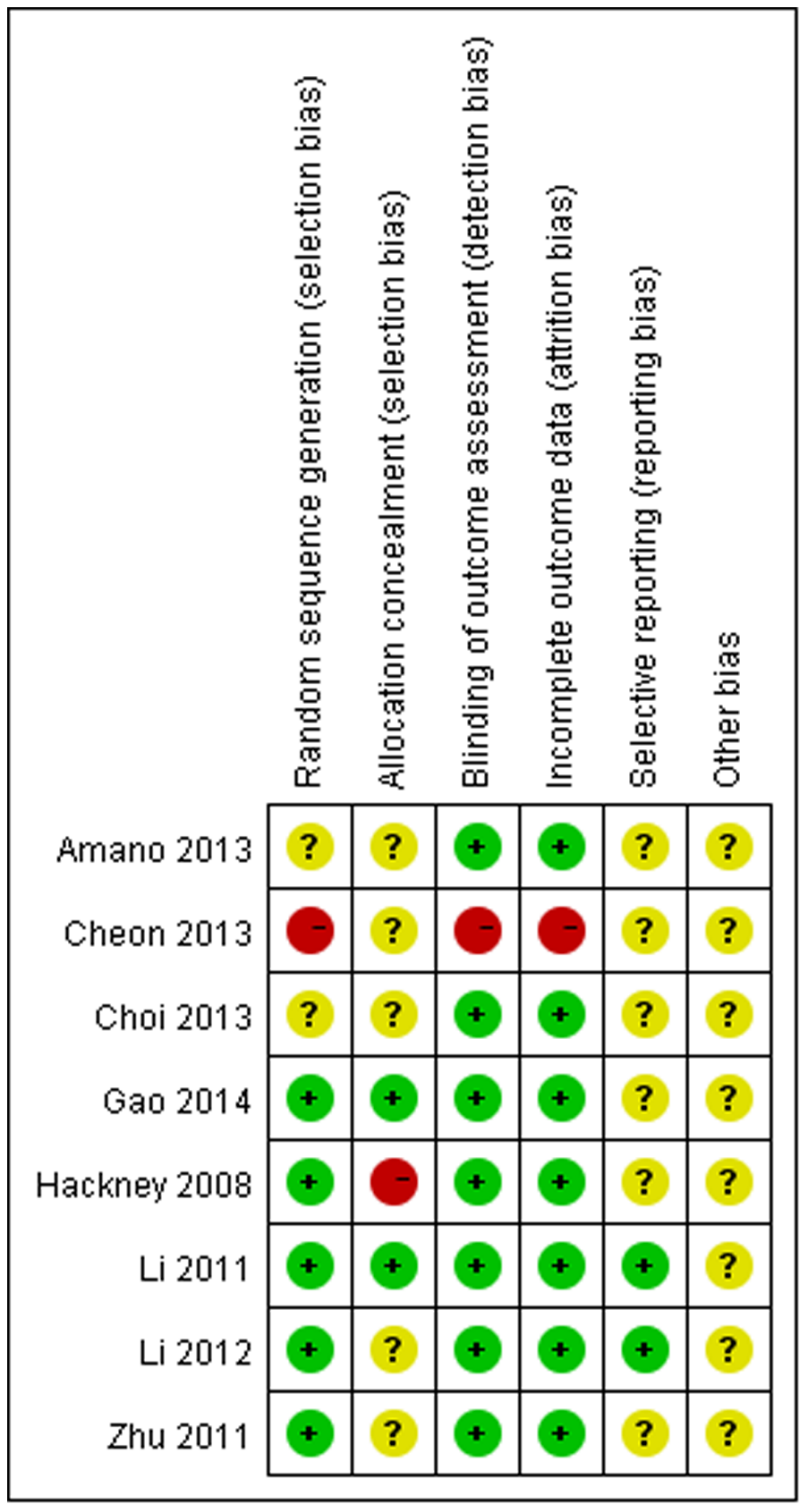
Risk of bias. Red (-): high risk of bias; Yellow (?): unclear risk of bias; Green (+): low risk of bias.

The risk of bias in the eligible studies was summarized in Figure 2. Five of them employed adequate random sequence generation [9], [12]–[14], [16]. Two was unclear for not describe detailed randomized methods [10], [15], and one was at high risk due to no random sequence generation[17]. Only two trials employed adequate allocation methods [13], [16]. Five studies did not describe the method for generating the allocation sequence [9], [10], [14], [15], [17]. And one study was considered to be high risk in allocation concealment because the authors were not blinded to group assignment [12]. While most studies reported blinding of outcome assessment [9], [10], [12]–[16], the evaluator was not blinded to group assignment only in one trial [17]. In incomplete outcome, one study was at high risk due to high drop-out rate [17], and others were at low risk [9], [10], [12]–[16]. Most of included studies were unclear in selective reporting due to without the study protocols [10], [12], [14]–[17]. Only two were considered to be low risk in this item [9], [13]. Other bias in all included studies was unclear because more detailed information was obtained from the primary authors.

### The Effect of Tai Chi for PD

#### Motor function

**Figure 3:**
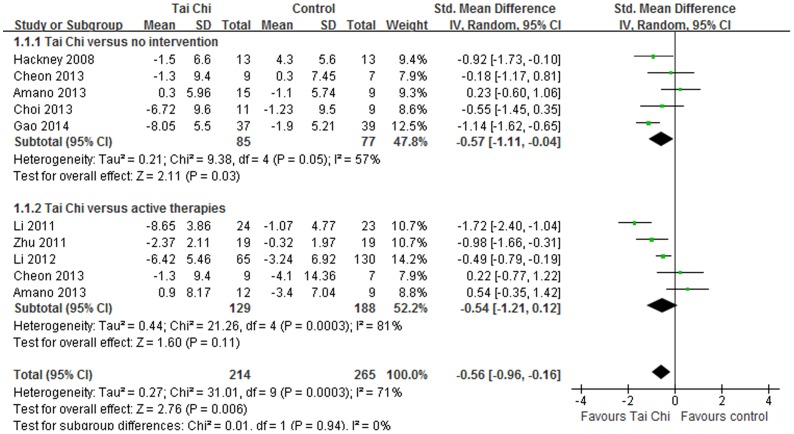
Forest plot showing the effect of Tai Chi on motor function in individuals with Parkinson's disease.

All included studies evaluated the effect of Tai Chi on motor function in individuals with PD by UPDRS III [9], [10], [12]–[17]. The aggregated results suggested that Tai Chi showed beneficial improvements on motor function in patients with PD (SMD, −0.57; 95% CI −1.11 to −0.04; p = 0.03, Figure 3) [10], [12], [15]–[17]. But the aggregated results did not support or refute the value of Tai Chi on motor function compared with other active therapies (SMD, −0.54; 95% CI −1.21 to 0.12; p = 0.11, Figure 3) [9], [10], [13], [14], [17].

#### Balance

**Figure 4:**
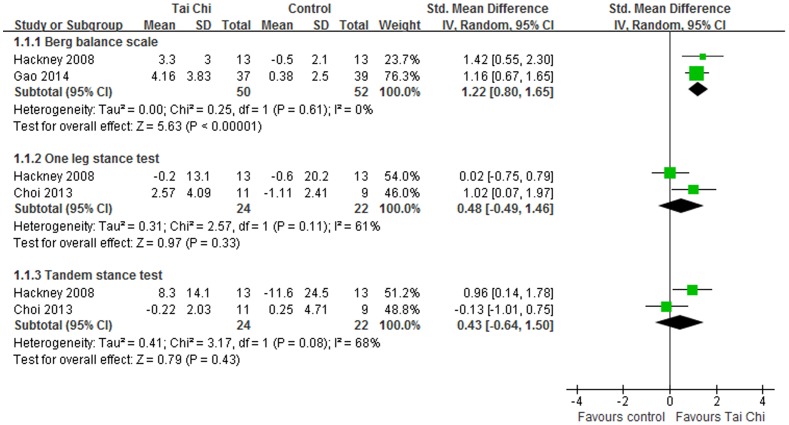
Forest plot showing the effect of Tai Chi on balance in individuals with Parkinson's disease compared with no intervention.

**Figure 5:**
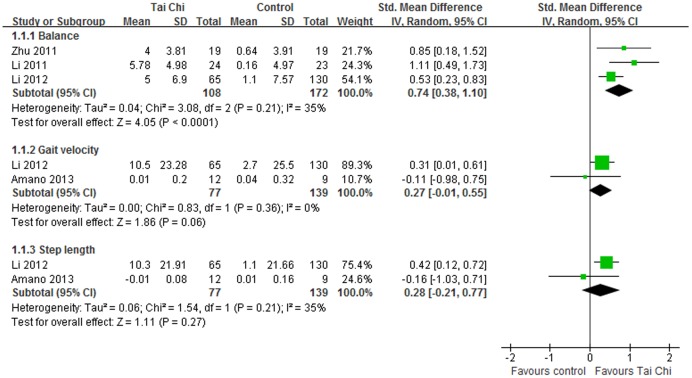
Forest plot showing the effect of Tai Chi on balance, gait velocity, step length in individuals with Parkinson's disease compared with other active therapies.

In eligible studies, balance was evaluated using BBS, TST, OLST and FRT. For BBS, the aggregated results supported that Tai Chi was effective in improving balance for individuals with PD (SMD, 1.22; 95% CI 0.80 to 1.65; p<0.00001, Figure 4) [12], [16]. And Tai Chi also showed better improvements on BBS compared with other active therapies (SMD, 0.74; 95% CI 0.38 to 1.10; p<0.0001, Figure 5) [9], [13], [14]. However, the meta-analyses suggested that Tai Chi did not show effective improvements on OLST (SMD, 0.48; 95% CI −0.49 to 1.46; p = 0.33, Figure 4) [12], [15] or TS (SMD, 0.43; 95% CI −0.64 to 1.50; p = 0.43, Figure 4) [12], [15].

#### Gait

**Figure 6:**
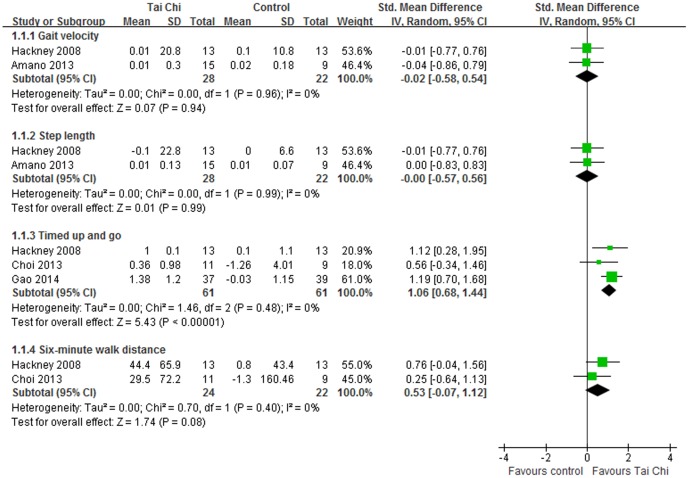
Forest plot showing the effect of Tai Chi on gait function in individuals with Parkinson's disease compared with no intervention.

Four studies assessed the gait in patients with PD compared with no intervention or active therapies. Tai Chi did not show beneficial performance in gait velocity (SMD, −0.02; 95% CI −0.58 to 0.54; p = 0.94, Figure 6) [10], [12] or step length (SMD, −0.00; 95% CI −0.57 to 0.56; p = 0.99, Figure 6) [10], [12]. Compared with active therapies, Tai Chi did not either outperform other active therapies in gait velocity (SMD, 0.27; 95% CI −0.01 to 0.55; p = 0.06, Figure 5) [9], [10] nor step length (SMD, 0.28; 95% CI −0.21 to 0.77; p = 0.27, Figure 5) [9], [10]. Gait endurance was assessed by 6-MWD. And Tai Chi did not show beneficial effects on improving gait endurance in patients with PD (SMD, 0.53; 95% CI −0.07 to 1.12; p = 0.08, Figure 6)[12], [15].

Functional mobility was assessed using TUG in 4 eligible studies [9], [12], [15], [16]. Three of them were included in the meta-analysis comparing Tai Chi with no intervention [12], [15], [16]. The aggregated results suggested that Tai Chi should be beneficial in improving functional mobility in individuals with PD (SMD, 1.06; 95% CI 0.68 to 1.44; p<0.00001, Figure 6) [12], [15], [16]. What's more, Li and colleagues reported that participants in Tai Chi group had better performance on TUG compared with ones in the stretching group [9].

### Adverse Events

No major adverse events were noted in included studies. Only Li reported that there were four ankle sprain, one low back pain and one muscle soreness in Tai Chi exercise group [9].

## Discussion

This systematic review demonstrates that Tai Chi showed beneficial effects on motor function, balance and functional mobility in patients with PD. But there was no sufficient evidence to support or refute the value of Tai Chi on gait velocity, step length or gait endurance. Compared with other active therapies, Tai Chi only showed better effects in improving balance. What's more, there was no sufficient evidence in the follow-up effects of Tai Chi for PD.

It is claimed that Tai Chi is effective in improving motor flexibility and balance for parkinsonian populations [6], [18]. Clearly these claims were tested with eight clinical trials in our systematic review. In this review, most RCTs showed that the implementation of Tai Chi produced positive effects on balance and motor function in people with PD [9], [12]–[14], [16]. The included non-RCT also suggested that Tai Chi yielded better results for functional fitness [17].The strict inclusion criteria increased the confidence in our results. Only studies with detailed and validated data in outcome measures were eligible in our review for conducting meta-analyses. Therefore, our systematic review showed the objective evidence of Tai Chi for PD based on aggregated results. What's more, detailed subgroup analyses were performed because different control comparators address different questions. The no intervention control is intended to address the question: is Tai Chi an effective therapy for PD. The aggregated results suggested that Tai Chi should be a beneficial therapy in improving motor function, balance and functional mobility in patients with PD. And the meta-analyses of Tai Chi compared with other active therapies address the question of whether Tai Chi is more effective than other active therapies for PD. The results only suggested that Tai Chi should be more effective in improving balance.

Our positive results concur with those from the last systematic review [19]. The systematic review performed by Ni and his colleagues concluded that Tai Chi resulted in promising gains in mobility and balance for pateits with PD, and Tai Chi was safe for PD patients. In this review, however, there was only one study in some subgroup meta-analyses. It was not proper to conduct meta-analysis for which should be performed based at least on two independent studies. In one eligible study, two similar control groups should be combined to create a single pair-wise comparison according to Cochrane handbook, but this systematic review did not perform it. No major adverse events associated with Tai Chi were noted in most eligible studies, but definite conclusion that Tai Chi was safe among PD patients was not possible in Ni's review. What's more, two new RCTs of Tai Chi in improving motor function in patients with PD were included in our meta-analyses [16], [17]. So our systematic review provided stronger evidence of Tai Chi in improving motor function in patients with PD.

It would be probably that the objective evidence made our review different from the previous ones [5], [20], [21]. Lee and colleagues concluded that the evidence was insufficient to suggest that Tai Chi was an effective intervention for PD based on three RCTs [22]–[24], one non-RCT [25] and 3 uncontrolled clinical trials [26]–[28] published from 1997 to 2007 [5]. And most of them were only conference abstracts without detailed essential information and validated data of outcomes [22]–[26]. And Toh's systematic review concluded that there were no firm evidence to support the effectiveness of Tai Chi in improving motor performance in patients with PD [21], but this qualitative systematic review only included 4 RCTs [9], [10], [12], [29], two single-arm intervention studies [27], [30] and two case reports [31], [32]. By contrast, more new studies were recruited in our review [9], [10], [12]–[17]. What's more, all full texts of seven RCTs [9], [10], [12]–[16] and one non-RCT [17] were published from 2008 to 2014. Another difference may be the detailed meta-analyses performed to investigate the effect of Tai Chi for PD in our systematic review. Hence our systematic review produced more confidence evidence of Tai Chi for PD.

People diagnosed PD often have impaired mobility or poor balance. The literature suggests that balance and mobility problems in people with mild PD may become apparent when more-complex coordination is required under challenging conditions [33], [34]. In Tai Chi exercise, the slow multiple direction movements and mind concentration could increase the strength of the lower limbs, attentiveness and the stability during weight shifting [35], [36]. These factors may contribute to the mobility and balance improvement. Although these improvements indicate that Tai Chi would be effective in promoting neuromuscular rehabilitation, the rationales behind the therapeutic effects in patients' motor control remain less understood and warrant further exploration.

Although this systematic review appeared to have positive results for PD population, it needs cautious interpretation because there is likely to be several limitations: (a) there was potential location bias due to language barrier, limited retrieving resources and publication bias. And the distorting effects of publication and location bias on systematic reviews are well documented [37], [38]; (b) there was one non-RCT in the meta-analysis of the motor function, but it did not affect the aggregated results. What's more, it also contributed valuable information to the evidence of Tai Chi for PD, especially on motor function; (c) our aggregated results may be affected by styles and dosing parameters of Tai Chi in eligible studies, such as different styles (Yang-style, Wu-style, 24-short form, etc.), duration (time of each Tai Chi), frequency (sessions of Tai Chi per week); (d) there were less eligible studies in some subgroups meta-analyses due to strict eligibility criteria in our review. It may influence aggregated results, but low eligibility criteria would generate more doubtful results; (e) the evaluation on the follow-up effect of Tai Chi for PD was insufficient in eligible studies. So the current results should be interpreted with caution and future studies should pay more attention to the follow-up effect; (f) although no major adverse events associated with Tai Chi were noted in included studies, definite conclusions were not possible. It only can be assumed that Tai Chi is a therapeutic option with low risk of injury.

## Conclusion

This systematic review showed the positive evidence that Tai Chi had beneficial effects in improving motor function, balance and functional mobility in people with PD. But there was insufficient evidence to support or refute the value of Tai Chi on gait velocity, step length or gait endurance. Compared with other active therapies, Tai Chi only showed better effects in improving balance. We recommend that Tai Chi exercise should be implemented into clinical practice as a complementary and alternative therapy for PD, particularly when the aim is to improve motor function and balance in patients with PD. However, more RCTs with long follow-up are warrant to confirm the current findings of Tai Chi for PD.

## Supporting Information

Checklist S1 **PRISMA checklist.**
(DOC)
